# Medical Students' and Residents' preferred site characteristics and preceptor behaviours for learning in the ambulatory setting: a cross-sectional survey

**DOI:** 10.1186/1472-6920-4-12

**Published:** 2004-08-06

**Authors:** Karen W Schultz, John Kirby, Dianne Delva, Marshall Godwin, Sarita Verma, Richard Birtwhistle, Chris Knapper, Rachelle Seguin

**Affiliations:** 1 Department of Family Medicine, Queen's University, 220 Bagot St., PO Bag 8888, Kingston, ON, Canada, K7L 5E9; 2 Faculty of Education, Queen's University, 511 Union St., Kingston, ON, Canada, K7M 5R4; 3 Department of Psychology, Queen's University Kingston, ON, Canada, K7L 3N6

## Abstract

**Background:**

Medical training is increasingly occurring in the ambulatory setting for final year medical students and residents. This study looks to identify if gender, school, level of training, or speciality affects learner's (final year medical students and residents) preferred site characteristics and preceptor behaviours for learning in the ambulatory setting.

**Methods:**

All final year medical students and residents at the five medical schools in Ontario (N = 3471) were surveyed about the site characteristics and preceptor behaviours most enhancing their learning in the ambulatory setting. Preferred site characteristics and preceptor behaviours were rank ordered. Factor analysis grouped the site characteristics and preceptor behaviours into themes which were then correlated with gender, school, level of training, and speciality.

**Results:**

Having an adequate number and variety of patients while being supervised by enthusiastic preceptors who give feedback and are willing to discuss their reasoning processes and delegate responsibility are site characteristics and preceptor behaviours valued by almost all learners. Some teaching strategies recently suggested to improve efficiency in the ambulatory teaching setting, such as structuring the interview for the student and teaching and reviewing the case in front of the patient, were found not to be valued by learners. There was a striking degree of similarity in what was valued by all learners but there were also some educationally significant differences, particularly between learners at different levels and in different specialities. Key findings between the different levels include preceptor interaction being most important for medical students as opposed to residents who most value issues pertaining to patient logistics. Learning resources are less valued early and late in training. Teaching and having the case reviewed in front of the patient becomes increasingly less valued as learners advance in their training. As one approaches the end of ones' training office management instruction becomes increasingly valued. Differences between specialities pertain most to the type of practice residents will ultimately end up in (ie: office based specialties particularly valuing instruction in office management and health care system interaction).

**Conclusions:**

Preceptors need to be aware of, and make efforts to provide, teaching strategies such as feedback and discussing clinical reasoning, that learners have identified as being helpful for learning. If strategies identified as not being valued for learning, such as teaching in front of the patient, must continue it will be important to explore the barriers they present to learning. Although what all learners want from their preceptors and clinic settings to enhance their learning is remarkably similar, being aware of the educationally significant differences, particularly for learners at different levels and in different specialities, will enhance teaching in the ambulatory setting.

## Background

"The ideal preceptor should be like Captain Picard from Star Trek, who has a good grasp of situations but lets his subordinates push themselves to their limits without interfering/imposing his views and methods"! (survey comment)

Medical care is being delivered primarily in the ambulatory setting in an increasing number of specialties. Since learning is best done contextually [1,2] it is appropriate and necessary that medical training also increasingly occur in the ambulatory setting. Theory suggests that trainees at different levels [3-8] and in different specialties [3,6,9-11] may have different learning needs. Students early in their training may be looking to be taught certainties about facts and concepts, corresponding to Perry's concept of simple dualism, ie; right versus wrong, and to not find it helpful to be engaged in discussions about "softer" emotional and social issues [5]. Stritter found first year residents preferred being told what to do, whereas higher-level residents preferred more autonomy and more explanations from their preceptors [7]. Work looking at learning styles in different specialities has been mainly based on Kolb's work, who outlined four different learning styles, and suggests those in different specialities learn differently (ie: surgeons learn best by hands-on practical application of ideas [11], while pathologists learn best using abstract theoretical models [10]).

An article by Kernan [12] outlined site accommodations and preceptor behaviours that third year medical students felt facilitated their learning during a one-month ambulatory internal medicine rotation. A pilot project at our institution asking first year family medicine residents to rank Kernan's study items found differences between the two groups. What was not clear was if these differences were due to school attended, level of training or specialty. Given that trainees at all levels and in all specialties are increasingly being trained in the ambulatory setting, it seemed important to understand if there truly are differences between different types of students in what is perceived as being most helpful for learning. If differences are identified it will then be important to study whether adjusting to these differences actually improves learning.

We surveyed all final year medical students and residents in Ontario about the site characteristics and preceptor behaviours that they find most enhance learning in the ambulatory setting and determined if these were related to demographic factors, level of training or residency program. Implications for teaching in the ambulatory setting are explored based on these results.

## Methods

All medical students (n = 532) and residents (n = 2939) at the five medical schools in Ontario were surveyed using a four part questionnaire which collected information on demographics, preferred site characteristics, preferred preceptor behaviours, and approaches to learning and perceptions of learning climate. Questions for the site characteristics and preceptor behaviours included previously validated questions [12-15] and questions believed to be important by study group consensus. The approaches to learning and perceptions of learning climate questionnaire was validated by Kirby et al [16] and is not reported here.

Students rated 24 site characteristics and 38 preceptor behaviours on a Likert scale from 1 (very important for learning) to 5 (not at all important for learning) or D (detrimental for learning). Within each section they indicated the five most important and 5 most unimportant or detrimental items for learning. A section for general comments was included at the end of the survey. The survey was piloted with a group of Queen's University residents and final year medical students checking for ambiguity and content. Ethical approval was granted by the Queen's University General Research Ethics Board. To ensure privacy for their students schools requested that the questionnaires be addressed by their own undergraduate and postgraduate offices. Coded questionnaires were thus sent with student's names and bulk mailed to the undergraduate and postgraduate medical schools who then addressed and forwarded the questionnaires to their final year medical students and residents. Entry into a draw for a Personal Digital Assistant or equivalent monetary prize was offered for completed surveys. Non-responders were identified by a lack of a returned coded questionnaire. Two subsequent mailings were sent to the non-respondents through their schools' undergraduate or postgraduate office. In addition an email reminder was sent to everyone between the second and third mailing.

Data were analyzed using SPSS for Windows, version 11.0 [17]. A systematic effort to look for out-of-range data was conducted by doing frequency distributions for each of the variables, identifying out-of-range entries and correcting the errors by going back to the original data sheets. Double entry data assessment was not done. Frequency distributions for demographic factors, valued site characteristics, and preceptor behaviours were compiled. Counts were derived for each site characteristic or preceptor behaviour by calculating percentages of respondents giving the item a score of 1 or 2 on the Likert scale. Detrimental items were tallied from the frequency data. Factor analysis of the site characteristics and preceptor behaviours was carried out. Cronbach alpha coefficients were calculated for the identified factors. (Factor analysis is a means of reducing a large number of items to a smaller, more manageable number of dimensions, based on the ways in which the items correlate with each other. Cronbach alpha coefficients can then be calculated for internal consistency of the scales based on the identified factors. The resulting factors/scales need to be interpreted, but may provide a view of underlying constructs that are responsible for the observed variables and their correlations. Both the choice of the number of factors to extract and the interpretation of the factors/scales are matters of interpretation [18].) Counts (derived by calculating percentages for each item ranked 1 or 2 on the Likert scale) were generated for gender, school, level of training and residency for each factor. The Post-Graduate Year 2 (PGY2) group had an additional, possibly confounding feature, containing a large number of family medicine residents, who would be at the end of their training, instead of half way through their training like the remainder of the group. A subanalysis was done on the level of training data removing family medicine residents from the PGY2 data to analyse the impact of this on preferred site characteristics and preceptor behaviours. Initial data interpretation for residency used 11 residencies. Residencies were then collapsed into five groups (medicine; family medicine, paediatrics, psychiatry; lab/path, radiology; surgery, emergency, ob/gyn; and intensivists,anaesthetists) based on similarity of practice patterns. Logistic regression analysis[17] was used to compare gender, school, level of training and residency with respect to the site characteristic and preceptor behaviour factors. Each independent variable (ie: gender, school, level of training and residency grouping) was entered individually into a regression procedure as a categorical variable and the proportion of positive responses (1 or 2 on the Likert scale) for each level of the variable was compared to the proportion of positive responses in the full sample.

## Results

Survey response was 48% (1642/3430). Of these 44 had not worked in an ambulatory setting and so were eliminated from further analysis (N = 1598). The demographics of the five medical schools are listed in Table 1. Demographic characteristics of responders are shown in Table 2. Comparisons to all Ontario and Canadian clerks and residents revealed more women, junior residents, McMaster and Family Medicine residents and fewer PGY6-fellows and Toronto trainees responded.

**Table 1:** Demographics of the five medical schools

**School**	**City Size**	# final year medical students (2001–02)*	# residents (2001–02)*
Queen's University	113,000	71	248
University of Toronto	4,700,000	167	1268
University of Western Ontario	432,000	98	353
University of Ottawa	823,000	85	398
McMaster University	662,000	103	396

* Data from Association of Canadian Medical Colleges

**Table 2:** Demographics of Study Group

**Demographic**	**Study Numbers N (%)**
**Gender**	N = 1642
Male	805 (49)
Female	837 (51)
**Level of Training**	N = 1641
Clerks	279 (17.0)
First year residents	377 (23.0)
Second year residents	366 (22.3)
Third year residents	231 (14.1)
Fourth year residents	165 (10.1)
Fifth year residents	185 (11.3)
Sixth and above year residents including Fellows	38 (2.3)
**University**	N = 1642
Queen's U.	172 (10.5)
U. of Toronto	611 (37.3)
U. of Western Ontario	243 (14.8)
Ottawa U.	296 (18.0)
McMaster U.	317 (19.3)
**Mean Age Residents**	29.9
**Training Program**	N = 1356*,**
Medicine	298 (22.0)
Family Medicine	351 (25.9)
Paediatrics	100 (7.4)
Surgery	226 (16.7)
Psychiatry	104 (7.7)
Radiology	56 (4.1)
Intensivists	7 (0.5)
Anaesthesia	102 (7.5)
Laboratory	24 (1.8)
Obstetrics/Gynaecology	65 (4.8)
Emergency	23 (1.7)

*N = total number-clerks **7 not specified

The rank ordering of site characteristics and preceptor behaviours, including missing data and number judging an item to not only be unhelpful but detrimental for learning are shown in Tables 3 and 4. The five most and five least important items for learning essentially matched the rank ordering and thus are not separately reported.

**Table 3:** Ranking of Site Characteristics

**Rank**	**Question**	**Number saying important to learning (%)**	**Number not answering question**	**Number saying detrimental for learning (%)**
1	Effective teachers	1569 (98.4)	4	3 (0.2)
2	Opportunity to see patients independently	1592 (97.3)	6	0
3	Opportunity to see a large variety of patients	1511 (94.8)	4	1 (0.1)
4	Opportunity to see an adequate number of patients	1496 (93.9)	5	2 (0.1)
5	Preceptors readily available	1490 (93.5)	4	2 (0.1)
6	Opportunity to do procedures	1357 (85.3)	8	3 (0.2)
7	Readily available examination room	1348 (84.8)	9	1 (0.1)
8	Opportunity to see patients in follow-up visits	1275 (80.1)	6	1 (0.1)
9	Opportunity to observe preceptor if desired	1239 (77.8)	6	0
10	Opportunity to interact with consultants and/or referring doctors	1227 (77.1)	7	0
11	Block rotation	1094 (68.8)	7	3 (0.2)
12	Efforts to meet objectives made by preceptor	1059 (66.5)	5	1 (0.1)
13	Teaching of medical record keeping skills	956 (60.0)	4	0
14	Computer learning resources available in the clinic	947 (59.4)	3	0
15	Orientation to the practice	937 (59.0)	11	1 (0.1)
16	Teaching of time management skills	904 (56.7)	3	3 (0.2)
17	Teaching of office management skills	872 (54.7)	4	2 (0.1)
18	Clearly defined site objectives for the rotation	843 (52.9)	5	4 (0.2)
19	Library resources available in the clinic	778 (48.8)	4	0
20	Existence of a site-coordinator	762 (48.4)	22	2 (0.1)
21	Longitudinal/horizontal rotation	603 (38.4)	29	42 (2.7)
22	Limited number of preceptors	443 (27.9)	13	233 (14.7)
23	Presence of other trainees in the clinic	432 (27.1)	4	74 (4.6)
24	Close proximity of clinic to campus	366 (23.0)	8	5 (0.3)

**Table 4:** Preceptor Behaviours Ranking

**Rank**	**Question**	**Number saying important for learning (%)**	**Number not answering question**	**Number saying detrimental for learning (%)**
1	Is open to questions	1540 (96.7)	5	1 (0.1)
2	Gives constructive feedback	1522 (95.6)	6	1 (0.1)
3	Demonstrates enthusiasm for teaching	1515 (95.1)	5	1 (0.1)
4	Reviews differential diagnoses	1507 (94.6)	5	0
5	Delegates appropriate responsibility for patient care	1491 (93.7)	7	1 (0.1)
6	Gives timely feedback	1445 (90.7)	5	0
7	Has a strong command of his or her specialty	1433 (90.1)	7	2 (0.1)
8	Discusses clinical topics in an organized way	1416 (88.9)	5	0
9	Makes student feel like a valued member of the practice	1407 (88.3)	5	1 (0.1)
10	Identifies and responds to student's specific learning needs	1398(87.9)	8	1 (0.1)
11	Discusses own clinical reasoning processes	1396 (87.3)	8	1 (0.1)
12	Asks for students' ideas before giving own	1372 (86.1)	5	0
13	Discusses clinical topics concisely	1361 (85.5)	6	1 (0.1)
14	Demonstrates a caring attitude towards students	1347 (84.6)	5	1 (0.1)
15	Sets time aside to discuss topics unable to be discussed during busy clinics	1340 (84.3)	10	4 (0.3)
16	Provides a role model of professional behaviour	1327 (83.5)	8	0
17	Asks students differing complexities of questions	1302 (81.8)	6	3 (0.2)
18	Welcomes differing points of view	1294 (81.3)	7	2 (0.1)
19	Demonstrates a caring attitude towards patients	1279 (80.3)	5	0
20	Facilitates student's participation in follow-up care	1263 (79.4)	7	0
21	Teaches physical examination	1218 (76.8)	13	3 (0.2)
22	Monitors quality of the rotation	1216 (76.4)	6	1 (0.1)
23	Seeks to understand student's ideas	1186 (74.5)	6	0
24	Suggests relevant reading	1172 (73.6)	5	0
25	Connects new ideas to existing knowledge	1149 (72.4)	10	0
26	Defines student's role	1087 (68.4)	8	3 (0.2)
27	Provides a role model of a balance between personal and professional life	1079 (67.9)	9	0
28	Teaches appropriate use of health care resources	1072 (67.4)	8	0
29	Teaches use of community resources	1005 (63.2)	7	0
29	Demonstrates effective interactions with support staff	1005 (63.2)	9	0
30	Observes clinical interactions directly	966 (60.8)	8	7 (0.4)
31	Teaches communication skills	940 (59.2)	10	2 (0.1)
32	Discusses limitations of his or her own knowledge	899 (56.5)	7	1 (0.1)
33	Provides background on patients before students sees patient	602 (37.8)	5	36 (2.3)
34	Outlines specific task(s) to be done during a clinical encounter	595 (37.5)	12	30 (1.9)
35	Teaches in the patient's presence	429 (27.1)	14	116 (7.3)
36	Focuses on one teaching theme per clinic	348 (21.9)	9	71 (4.4)
37	Reviews case in the patient's presence	281 (17.7)	10	242 (15.1)

Six factors, accounting for 55 % of the variance for the 24 site characteristics, and 7 factors, accounting for 54% of the variance for the 38 preceptor behaviours, were identified (Tables 5,6). Labels describing the factors were decided by group consensus among the researchers. 7 items that failed to load on any factor were eliminated from the analysis. The site characteristic factors were office management, patient logistics, objectives, learning resources, clinic set-up and preceptor interaction. The preceptor behaviour factors were professional role modeling, teaching, learning climate, feedback, direction, patient presence and health care system interaction. Cronbach alpha coefficients for the factors identified in the factor analysis ranged from 0.52 to 0.83.

**Table 5:** Factor Analysis makeup for Site Characteristics

**Factor**	**Items making up factor**	**Factor Loading**	**Alpha Analysis**
**Office Management**	Teaching of time management skills	.832	.62
	Teaching of medical record keeping skills	.760	
	Teaching of office management skills	.746	
**Patient Logistics**	Opportunity to see an adequate number of patients	.766	.69
	Opportunity to see a large variety of patients	.542	
	Opportunity to see patients independently	.538	
	Readily available examination room	.473	
	Opportunity to see patients in follow-up visits	.442	
**Objectives**	Clearly defined site objectives for the rotation	.806	.53
	Efforts to meet objectives made by preceptor	.776	
**Learning Resources**	Library resources available in the clinic	.794	.60
	Computer learning resources available in the clinic	.756	
**Clinic Set-up**	Close proximity of clinic to campus	.442	.55
	Presence of other trainees in the clinic	.418	
	Existence of a site co-coordinator	.386	
	Longitudinal/horizontal rotation	.364	
	Orientation to the practice	.342	
**Preceptor Interaction**	Effective teachers	.514	.55
	Preceptors readily available	.506	
	Opportunity to observe preceptor if desired	.491	

**Table 6:** Factor analysis for Preceptor Behaviours

**Factor**	**Items Making Up Factor**	**Factor Loading**	**Alpha Analysis**
**Professional Role Modeling**	Provides a role model of professional behaviour	.681	.79
	Demonstrates effective interactions with support staff	.565	
	Provides a role model of a balance between personal and professional life	.557	
	Teaches communication skills	.526	
	Discusses limitations of his or her own knowledge	.500	
	Discusses own clinical reasoning processes	.426	
**Teaching**	Discusses clinical topics in an organized way	.739	.82
	Discusses clinical topics concisely	.650	
	Suggests relevant reading	.462	
	Identifies and responds to student's specific learning needs	.390	
	Is open to questions	.365	
	Asks students differing complexities of questions	.362	
	Has a strong command of his or her area of specialty	.340	
	Asks for students' ideas before giving own	.334	
	Sets time aside to discuss topics unable to be discussed during busy clinics	.323	
	Monitors quality of the rotation	.301	
**Learning Climate**	Makes student feel like a valued member of the practice	.613	.83
	Demonstrates a caring attitude towards students	.591	
	Seeks to understand student's ideas	.563	
	Demonstrates a caring attitude towards patients	.512	
	Demonstrates enthusiasm for teaching	.365	
	Welcomes differing points of view	.328	
	Facilitates student's participation in follow-up care	.301	
**Feedback**	Gives constructive feedback	.730	.73
	Gives timely feedback	.709	
	Reviews differential diagnosis	.473	
**Direction**	Outlines specific task(s) to be done during a clinical encounter	.588	.69
	Focuses on one teaching theme per clinic	.507	
	Provides background on patients before student sees patient	.447	
	Teaches physical examination	.432	
	Defines student's role	.404	
**Patient Presence**	Teaches in the patient's presence	.759	.77
	Reviews case in the patient's presence	.720	
**Health Care System Interaction**	Teaches use of community resources	.531	.82
	Teaches appropriate use of health care resources	.516	

**Figure 1:**
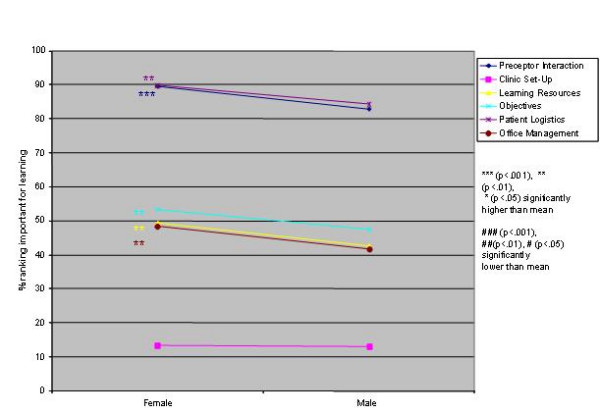
Gender and site characteristics

**Figure 2:**
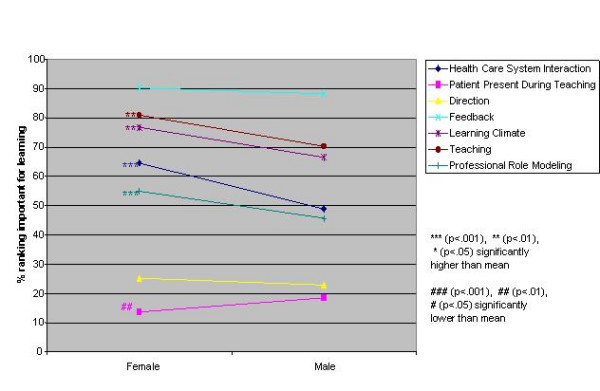
Gender and preceptor behaviours

**Figure 3:**
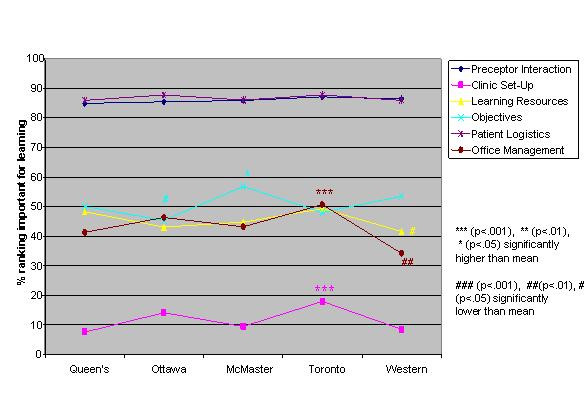
School and site characteristics

**Figure 4:**
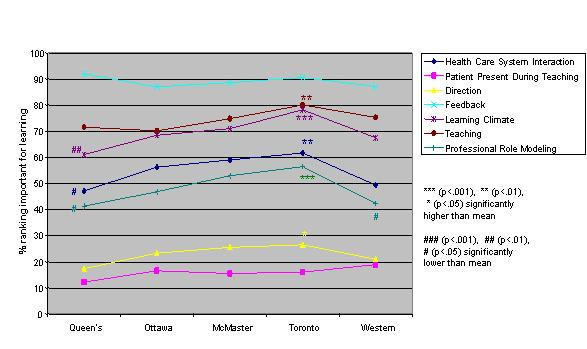
School and preceptor behaviours

**Figure 5:**
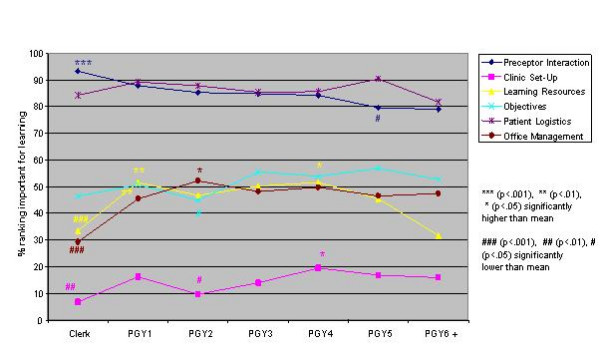
Training level and site characteristics

**Figure 6:**
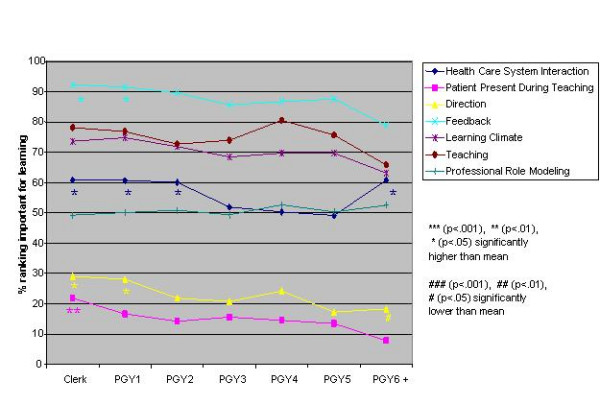
Training level and preceptor behaviours

**Figure 7:**
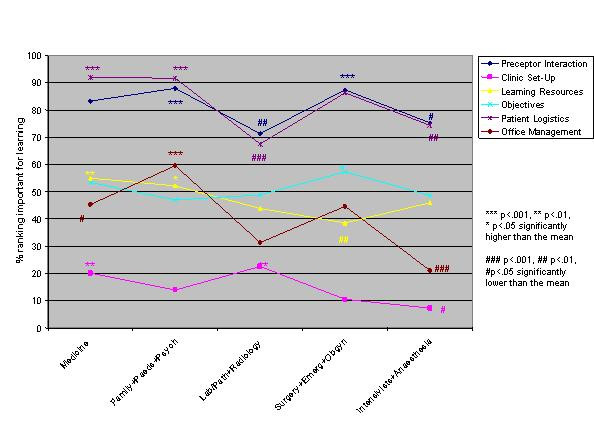
Residency and site characteristics

**Figure 8:**
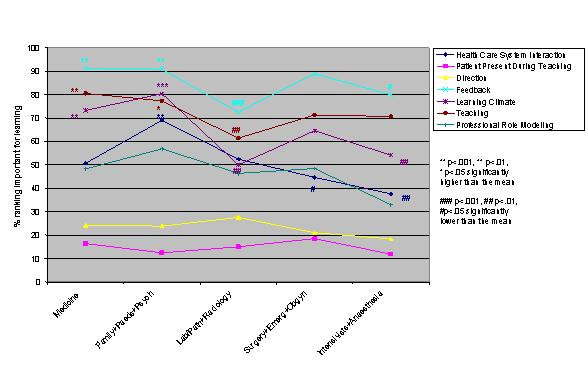
Residency and preceptor behaviours

Logistic regression analysis of the independent variables revealed striking similarities, but some significant differences, in valued site characteristics and preceptor behaviours for male and female students and those in different schools, at different levels of training and in different residencies (Figures 1,2,3,4,5,6,7,8). Similarities are evident by mainly flat, nonintersecting lines on the graphs indicating similar percentages of subgroups of learners valuing a factor and similar relative valuing of factors respectively. Differences are apparent where lines intersect within a graph and/or percentages are statistically higher (indicated by *, **, ***) or lower (#, ##, ###) than the means.

Male and female residents rank ordered all site characteristics and preceptor behaviours identically. Women ranked all factors, with the exception of teaching in the patient's presence, higher than men, usually significantly so (Figures 1,2).

Across schools the rank ordering of factors was identical with the exception of Toronto and Ottawa ranking office management instruction higher than the other schools. Toronto valued six factors significantly more than the other schools, Queen's and Western ranked three and two items respectively significantly less than the other schools (Figures 3,4).

Across levels the only difference in rank ordering was clerks ranking preceptor interaction as the most important site characteristic whereas all other groups ranked patient logistics as most important. Those at the beginning and end of their training valued having learning resources available less than all other levels. Clerks were most different from all the other levels in what they valued or did not value (indicated by the number of * and # for this group) (Figures 5,6). Subanalysis of the PGY2 data removing family medicine residents significantly decreased the importance of office management and health care system interaction instruction (52.2% of all PGY2's rated office management instruction important versus 44.8% removing family medicine residents, and 60.1% of all PGY2's rated health care system interaction instruction important versus 50.5% removing family medicine residents). Residency groups again showed mainly similarities in rank ordering, the exceptions being the family medicine/paediatrics/psychiatry group ranking office management and learning climate higher, the lab/path/radiology group patient logistics and learning climate lower and the surgery/emergency/ob/gyn group health care system interaction lower than the rest. There were a large number of responses significantly different from the group averages throughout all the residency groups (Figures 7,8). Combining residencies into five groups lost only two pieces of information, that of ob/gyn residents being similar to the family medicine, paediatrics, psychiatry group in relatively highly valuing office management instruction and that of anaesthesia residents being similar to lab/path, radiology residents in relatively less valuing feedback, teaching, and learning climate than other groups.

## Discussion

The ambulatory teaching site characteristics most valued by clerks and residents are having an adequate number and variety of patients while being supervised by enthusiastic and available preceptors. These characteristics have been identified before and are well summarized by Bowen and Irby [19]. Little value is placed on having other trainees in the clinic despite social learning theory that suggests this enhances learning. Bowen [20] and Lesky [21] suggest that students learn by teaching and may feel less threatened asking questions that reveal a lack of knowledge of a fellow student than of a preceptor. Although what students value may not translate into effective learning, it is still important to understand why something is valued or not valued. Without reliable learning outcome measures perceived learning value is a proxy measure of learning effectiveness. Further studies should assess what learners do not like about having other trainees present.

Computer resources were more valued than books, likely reflecting a generation of learners who are comfortable accessing electronic information. Proximity of the clinic to university campus was unimportant. In contrast to other studies [22-24] we found block rotations were valued more than longitudinal rotations. Some programs, particularly Canadian Family Medicine programs, encourage longitudinal rotations to enhance the continuity of care experience. Merenstein et al [25] however recently reported there to be no difference in continuity of care provided by residents in longitudinal rotations. Exploration of the value of block versus longitudinal rotations is an area for further research.

Valued preceptor behaviours identified in this study are feedback by enthusiastic, open preceptors who are willing to discuss their reasoning processes and delegate responsibility. Recent studies report 3^rd ^year medical students to also value these preceptor behaviours[26,27]. Lesky and Borkan [21] suggest that pathogenesis and natural histories of disease can be learned from a variety of resources, including books and computers but problem solving, decision making and dealing with uncertainty are learned mainly from preceptors and practice. This study supports students' perceived value of these aspects and suggests them as priorities for teachers in ambulatory settings.

We have confirmed the value of feedback found in most studies [26,28-31] (a study by O'Malley [32] being the exception). As one respondent commented "constructive and honest feedback in a timely manner is by far the most important (item)". Feedback leads to positive learning outcomes. Cope [33] demonstrated that giving feedback to residents improved their patient satisfaction scores, which in turn has been correlated with improved patient outcomes[34]. Unfortunately this teaching behaviour is underutilized. Irby [1], in a review of studies, reports that feedback is given only 3–6% of the time (range 0–16%). This is an effective teaching behaviour that is valued by students and deserves high priority.

Meaningful feedback about many aspects of students' patient care is best based on direct observation[35]. Only 61% of our respondents actually value direct observation by their preceptors. Some of the reasons for more not valuing this may be similar to why students do not want to be taught in front of the patient (see next section). Since direct observation is a necessary component of good teaching it will be important to explore further why more students do not value this important preceptor behaviour.

A number of strategies have been suggested to improve efficiency in the ambulatory teaching setting including teaching in the patient's presence and preceptors directing tasks to be covered in the interview [36-38]. A significant proportion of our respondents rated reviewing the case and teaching in the patient's presence, structuring the interview by providing patient information background, outlining tasks to be done during the interview and focusing on one teaching theme per clinic not only to be unimportant for learning but detrimental. Kernan similarly found 3^rd ^year medical students to not value being taught in front of the patient[26]. Comments from students in this study give some indication why teaching in front of the patient is disliked ("it would undermine a therapeutic alliance with the student", "it gives a tense atmosphere more often than not", "...impairs free thinking of student because student feels inhibition in front of patients", "makes it difficult for students to ask questions, not wanting to scare/worry the patient"). Teaching however occurs within a larger context where providing background information on patients may be necessary for ongoing patient care and safety and to model continuity of care. Teaching in the patient's presence may be necessary for efficiency and maintaining a relationship between the preceptor and the patient.Further studies are needed to determine if explanation or teaching methods can overcome this aversion.

Analysis of the impact of gender, school, level of training or residency on valued site characteristics and preceptor behaviours revealed striking uniformity between the groups. There were some statistically significant differences between the groups, many of which do not appear to be educationally relevant, others which likely are important.

Male and female students rank ordered site and preceptor behaviour factors identically. It is of interest that female students ranked all factors, with the exception of teaching in the patient's presence, as being more important for learning than male students. The literature [39-41] suggests that women predominantly emphasize relationship issues, which may partially explain this finding. It would appear however with respect to the items surveyed that there are no gender-based educationally important differences in valued site characteristics and preceptor behaviours.

The five schools also essentially rank ordered the factors identically. One school did stand out from the others in frequently ranking factors significantly higher than the rest. This school is the largest of the five schools with the most trainees and teaching sites. It would be valuable to know the ratio of students to preceptors at the different schools. If this were high at the larger school, perhaps resulting in residents feeling relatively anonymous, it may partially explain why these students there particularly value factors like learning climate, professional role modeling and clinic set up.

Within level of training preceptor interaction is most important for clerks. This is the only group to rank this item more important than patient logistics. This may reflect the clerks' developmental stage of being eager to go beyond textbook lists and start to put clinical decisions into patient context–skills best learned by preceptor interaction. Learning resources are significantly less valued by those at either end of their training–clerks for perhaps the above reason and PGY6's/fellows presumably because they are confident in their theoretical knowledge. Beyond clerkship patient logistic factors usurp preceptor interaction as the highest ranked site characteristic. Becoming an expert clinician involves, in part, connecting disparate units of knowledge into networks [3,5,42]. This encapsulating of knowledge occurs when students learn with patients. The residents in this study recognize this, ranking seeing an adequate and large variety of patients independently as the most important site characteristic for their learning. Having objectives defined with efforts made to meet them was third in importance for most levels, superceding available learning resources, office management skills instruction, and clinic setup items. Office management instruction is relatively more important for PGY2's and those at the end of their training. Subanalysis of the PGY2 data removing family medicine residents who would be at the end of their training and leaving those in the middle of their training significantly decreased the importance of office management and health care system interaction instruction. Teaching these aspects thus seems most important for those at the end of their training. Directing the clinical encounter and teaching in the patient's presence is valued less as residents gain seniority and presumably identify themselves more as the patients' physicians. Increasing desire for autonomy and decreasing potential for undermining their relationship with the patient may be reasons for these trends.

Within almost all residencies patient logistics and preceptor interaction are the most valued site characteristics; feedback, teaching and learning climate the most important preceptor behaviours. Lab/path, radiology and anaesthesia residents value all these preceptor behaviours less than other residents. Arguably these are areas of medicine where decision making is more clear-cut without as much patient input, which may explain these results. Other significant differences between the specialties seem best explained by considering future practice ie: office-based specialties (paediatrics, psychiatry, family medicine) most valuing office management and health care system interaction instruction.

Strengths of this study are the large multi-institutional sample size (n = 1642) encompassing students at multiple levels in all specialties. The response rate (48%) limits the external validity of the result. A confounding factor within the level of training data set may be the variability in residency lengths as suggested by the subanalysis of the PGY2 data. Rather than years from graduation from medical school what seems to influence valued site characteristics and preceptor behaviours more are years from independent practice.

## Conclusions

"Software" (patient encounters and enthusiastic preceptors who delegate, give feedback and explain clinical reasoning) is valued more than "hardware" (clinic set-up, learning resources). All learners value the above preceptor behaviours; most do not value, and a significant number consider detrimental, having the structure of the patient encounter dictated to them and having the patient present during review and teaching. Future work is needed to explain why learners do not value these practices. Learners at all levels and in all specialties are strikingly similar in what they value from their preceptors and clinic sites for their learning. There are some differences between levels and residencies however that require consideration when teaching these different groups. Educationally significant differences within levels include preceptor interaction being paramount for medical students; patient logistics (adequate number and variety of patients seen independently and in follow-up) being second. The reverse is true for residents. Proportioning time accordingly deserves attention. The more senior the learner the more being taught or having the case reviewed in the patients' presence is not valued. Sensitivity to the patient-learner relationship is required if these practices are utilized but particularly so for more senior learners. Finally relevance not surprisingly dictates importance. Office management instruction is valued by those at the end of their training and those primarily in office-based specialties. Similarly office-based specialties appreciate instruction in health care system interaction. This study identifies preceptor behaviours and site characteristics valued by medical students and residents for their learning in the ambulatory setting. Further studies are needed to determine the effect of providing these valued site characteristics and preceptor behaviours on learning outcomes.

## Competing interests

None declared.

## Authors' contributions

KS conceived of the study, prepared the manuscript and participated in the conceptual planning and design of the study and data interpretation. JK, DD, and MG participated in the conceptual planning and design of the study, statistical analysis and data interpretation and manuscript revision. SV and RB contributed to the design of the study and manuscript revision. CK contributed to the design of the study. All authors read and approved the final manuscript. RS participated in the statistical analysis.

## Pre-publication history

The pre-publication history for this paper can be accessed here:


